# A pragmatic implementation and outcomes evaluation of the older persons emergency network acute outreach service (OPEN AOS) model utilising the integrated PRISM and RE-AIM framework: the OPEN AOS study protocol

**DOI:** 10.1186/s12877-025-06917-2

**Published:** 2026-01-13

**Authors:** Sharon Hodby, Denise Bunting, Catherine Moore, Emma Ballard, Evelyn Kang, Joshua Byrnes, Julia Crilly, Nadine E. Foster, Elizabeth Marsden

**Affiliations:** 1 Metro North Hospital and Health Service, Older Persons Emergency Network, T9 Caboolture Medical Hub, 124 Mc Kean Street, Caboolture, 4510 Australia; 2 Queensland Ambulance Service, Department of Health, Cnr Park & Kedron Park Roads, Kedron, QLD 4031 Australia; 3 Metro North Hospital and Health Service, T9 Caboolture Medical Hub, 124 Mc Kean Street, Caboolture, 4510 Australia; 4 School of Nursing, Midwifery and Social Work, Faculty of Health, Medicine and Behavioural Sciences, The University of Queensland, Statistics Unit, QIMR Berghofer, 300 Herston Road, Herston, St Lucia, QLD 4072 Australia; 5 Faculty of Health Science, Higher College Technology Abu Dhabi and School of Nursing and Midwifery, Abu Dhabi, United Arab Emirates; 6 Griffith University, Griffith School of Nursing and Midwifery, G16 Clinical Sciences 2, Level 2.15, Gold Coast, QLD 4215 Australia; 7 School of Medicine and Dentistry, Griffith University, Centre for Applied Health Economics, 170 Kessels Road, Brisbane, QLD Australia; 8 Department of Emergency Medicine, Gold Coast Hospital and Health Service, 1 Hospital Blvd, Southport, QLD 4215 Australia; 9 Surgical Treatment and Rehabilitation Service (STARS), STARS Education and Research Alliance, The University of Queensland and Metro North Health, 296 Herston Rd, Herston, QLD 4006 Australia; 10 School of Health and Rehabilitation Sciences, Faculty of Health, Medicine and Behavioural Sciences, The University of Queensland, Brisbane, QLD 4072 Australia; 11 Faculty of Medicine, University of Queensland, Brisbane, QLD Australia

**Keywords:** Geriatrics, Homes for the Aged, Emergency medical services, Delivery of healthcare, Evaluation studies/research, Healthcare Economics

## Abstract

**Background:**

Globally, the population aged 65 years and over is increasing and with this growth comes a rising risk of chronic diseases that often require emergency care. Older adults living in Residential Aged Care Facilities (RACFs) have increased healthcare utilisation including visits to Emergency Departments (EDs). EDs are busy, noisy environments poorly suited to meeting the care needs of older adults. Older adults attending EDs often receive delayed and fragmented care, unnecessary procedures and tests and are at greater risk of iatrogenic complications.

The Older Person’s Emergency Network Acute Outreach Service (OPEN AOS) based in Queensland, Australia, is a nurse-led, physician-supported model that provides ED substitution care to older adults living in RACFs. This paper outlines the OPEN AOS study protocol, which comprises three components and uses the integrated Practical, Robust, Implementation, and Sustainability Model (PRISM) and Reach, Effectiveness, Adoption, Implementation and Maintenance (RE-AIM) framework to guide a comprehensive evaluation.

**Methods:**

The study comprises a pragmatic multi-methods design, consisting of three discrete yet related components. Component 1 is a retrospective, quasi-experimental design comparing outcomes for patients seen in the RACF by the OPEN AOS clinical team (intervention group) to standard ambulance attendance in the RACF resulting in transport to ED care (control group) between 1 July 2022 and 30 June 2023. Component 2 is a retrospective economic evaluation of the OPEN AOS model measuring the incremental cost per patient of OPEN AOS care compared to standard ambulance attendance resulting in transport to ED care. Component 3 adopts a prospective qualitative approach to explore and understand the contextual factors influencing implementation with the aim of informing effective delivery and supporting the potential scale and spread of the OPEN AOS model.

**Discussion:**

The OPEN AOS model aims to deliver ED-substitution care in RACFs for older adults. This study will adopt a pragmatic approach to assess both outcomes and implementation processes, utilizing the integrated PRISM and RE-AIM frameworks for comprehensive evaluation. Findings will inform the design of similar models and guide health system decision-making for adopting approaches like the OPEN AOS model.

**Supplementary Information:**

The online version contains supplementary material available at 10.1186/s12877-025-06917-2.

## Background

Globally the sustained growth of the older population poses numerous challenges for both individuals and healthcare systems. By 2030 the world is predicted to have one billion older adults, accounting for 13% of the total population [1]. In Australia, the proportion of people aged 65 years and over will increase from 17% in 2022 to between 25% and 27% in 2071 [2]. As people age, they are more likely to have multiple chronic health conditions that require healthcare [3–5]. Between 2012 and 2022 Australia saw admissions into Residential Aged Care Facilities (RACFs) double [6]. The utilisation of Emergency Departments (EDs) also increased [7]. In 2017-18 patients aged 65 years and older accounted for 21.6% (*n* = 1,731,121) of the 8 million ED presentations and this increased to 23.3% (*n* = 2,105,259) of the 9 million presentations in 2023-24 [8, 9].

A systematic review of qualitative studies by O’Neill et al. (2015) highlights the complex factors influencing ED transfer decisions from RACFs. Key factors included poor care planning, limited access to timely medical reviews, inadequate staffing within RACFs, medico-legal risk mitigation and conflict among stakeholders regarding the necessity of transfer [10]. While the ED is uniquely designed to provide assessment and diagnostics for the prevention, treatment and management of the critically ill or injured, it is a suboptimal environment for the provision of care to the frail, older adult [11]. Older patients often have long lengths of ED stays [12, 13], and if from an RACF, can undergo unnecessary investigations and invasive interventions with an increased risk of developing iatrogenic complications if admitted [14]. Older adults often experience longer wait times to be offloaded from an ambulance and are more likely to exceed the benchmarked wait time be to seen by an emergency doctor [15–18].

Existing policies set standards of care for older people in EDs [19] and evidence from both ED-focused models [20–22] and supportive care models – locally and internationally – shows that while specialized ED care is important, transportation of many older patients to the ED could be avoided if supportive care models were available. These care models include enhanced primary care for improved surveillance and management of illness; advanced care directives; palliative care; and clinical pathways for specific disease management [23–27].

However, the coordination and continuity of care for the older adult remains paramount [28, 29]. There are key considerations when providing acute care to an individual in the RACF such as: ascertaining their goals of care; treatment beneficence; non-maleficence (e.g. avoiding futile treatment); and understanding the capacity to diagnose and manage care safely in the home [30]. Various medical problems can be effectively treated and managed in the resident’s home such as urinary tract infections and falls with minor injuries including skin tears or simple lacerations [31]. A recent systematic review of ED transfers determined up to 55% were potentially avoidable, meaning the resident was safe to remain in their home utilising alternative care pathways [32]. Additionally, some evidence suggests that RACF residents and their families have a general preference for treatment to be provided in their home environment [33].

A systematic review of 15 observational studies evaluating acute outreach models of care to people living in RACFs on ED transfer prevention, hospitalisation, safety, cost-effectiveness and experiences [34] highlighted the absence of evidence. The authors recommended that future cluster-randomised or quasi-experimental studies be undertaken to assess the effectiveness and safety of these new outreach models [34].

To support the provision of emergency care for residents in RACFs in Queensland Australia, a novel acute ED substitution outreach model called OPEN AOS (Older Persons Emergency Network Acute Outreach Service) was established. The overarching aim of this study is to evaluate the implementation and outcomes of this ED outreach model. This paper describes the protocol for the evaluation of the OPEN AOS model.

## Methods

### Study aims

The aims of the OPEN AOS model evaluation are to:

1. Describe and compare the effectiveness and safety of the OPEN AOS model with standard ambulance attendance resulting in transport to ED care (standard care).
2. Conduct an economic evaluation of the OPEN AOS model; and.
3. Explore and understand the contextual factors influencing implementation to inform delivery and support potential scale and spread of the OPEN AOS model.

## Setting

The study is set in the Metro North Hospital and Health Service (MNHHS) district in south-east Queensland, Australia [35]. In 2024, the MNHHS region had 87 RACFs (85 non-government) with 9417 RACF beds. The district is serviced by five public hospitals; four are classified as large or very large and one is classified as small. The EDs within each of the five hospitals vary in their level of service capability and capacity [36, 37].

## Control: standard ambulance model of care

In Queensland, prehospital emergency care is provided by the Queensland Ambulance Service (QAS), a single, state-wide, government-funded emergency ambulance service. Like other Australian and international ambulance services, primary triage of calls is completed by a non-clinical Emergency Medical Dispatcher to determine the nature and type of incident, and the response time criticality. All incidents are assigned as either: emergency (potentially life threatening – immediate response with lights and siren); urgent (acute – immediate response with no lights and siren); or non-emergency (time critical scheduled conveyance or routine) response. Incidents are subsequently assigned either an immediate dispatch response or are transferred for clinical review performed by a multidisciplinary team of experienced paramedics, medical practitioners, mental health clinicians, social workers and Registered Nurses (RNs). Following clinical review, patients are assigned the most appropriate health response, which may include a dispatch response or referral to equivalent care pathway, and/or virtual care (i.e., health care consultation provided via telehealth platform run by RNs and medical practitioners). The majority of QAS patients (over one million annually) are transported from the scene [38], and approximately 90.0% of transports are to public hospital EDs [39].

## Intervention: the OPEN AOS model

The OPEN AOS model provides emergency substitution care to older adults residing in RACFs located within the MNHHS catchment area. Nurse-led and physician supported, OPEN AOS commenced in January 2022 and became fully operational by June 2022. The service is based in office space adjacent to a MNHHS hospital and operates 12 h per day between the hours of 08:00–20:00, Monday to Sunday. The service aims to (i) decrease the time to treating emergency clinician and (ii) reduce the number of avoidable ambulance transports to ED for older adults living in RACFs. The OPEN AOS model is staffed by 3.0 full-time equivalent (FTE) Nurse Practitioners^1^ (NP); 1.0 FTE NP candidates (NPC)^2^; and 1.8 FTE RNs^3^ [40, 41]. The model is supported by 1.4 FTE Senior Medical Officers (SMO)^4^ [42, 43] and 0.8 FTE Senior Pharmacists 5 [44]. The service is funded by MNHHS. Detailed descriptions of the responsibilities of the OPEN AOS clinical roles are provided in Additional file 1.

Patients of OPEN AOS are older adults residing in RACFs experiencing acute illnesses or injuries. OPEN AOS clinicians provide emergency substitution care via telehealth or in person. Referrals originate from the ambulance service (either from paramedics attending the patient at the RACF or following secondary telephone triage by experienced ambulance service clinicians), General Practitioners (GPs), RACF staff and other local teams providing specialist sub-acute RACF consulting services. Each referral is reviewed by an OPEN AOS clinician who triages the case and determines suitability for OPEN AOS care. Patients not meeting eligibility criteria for the OPEN AOS remain in the care of ambulance services for secondary triage and receive standard clinical review, consideration of referral pathways and standard ambulance attendance.

## Study design

This pragmatic multi-methods study comprises three discrete but related components: concurrent retrospective quantitative data collection for Components 1 and 2, and prospective qualitative data collection for Component 3. The approach to quantitative and qualitative data collection enables a comprehensive examination of the model’s effectiveness and real-world application. Given the multi-methods design, integration of quantitative and qualitative data through triangulation will not be undertaken. The design is underpinned by the integrated Practical, Robust, Implementation and Sustainability Model (PRISM) and Reach, Effectiveness, Adoption, Implementation and Maintenance (RE-AIM) framework [45]. PRISM will support the identification and description of contextual factors impacting RE-AIM outcomes [45]. This study has been reviewed and approved by the Metro North Human Research Ethics Committee (HREC) B (ECOO168), approval number 102,923. Data storage, retention and disposal will be conducted in accordance with the National Health and Medical Research Council (NHMRC) guidelines [46].

Three components are planned to address the following research questions:

*Component 1*

- To what extent (if any) do patient characteristics (such as age, sex, and diagnostic classification^6^) and outcomes differ between OPEN AOS care and standard care (i.e. ambulance attendance with transport to ED care)?
- Specifically:

4. Primary Outcome: Time to be seen by treating clinician, defined as the amount of time in minutes from the initial contact with QAS to the time seen by either (i) the OPEN AOS clinician (intervention group) or (ii) the ED medical officer (control group).
5. Secondary Outcomes:
  6. Length of care episode, defined as the amount of time in hours from initial contact with QAS to discharge of care.
  7. Ambulance transport to ED care: defined as the proportion of OPEN AOS patients requiring transport to ED for definitive care.
  8. ED re-presentation for any cause within 48 h and 28 days of discharge.
  9. Death within 48 h of discharge.
  10. Clinical incident^7^ [47] data, defined as clinical incidents that are reported by MNHHS staff that occur within 48 h of discharge.

*Component 2*

Compared to standard care, what is the incremental cost (saving) per person of the OPEN AOS model? 

*Component 3*

How do contextual factors influence implementation and potential future scale and spread of the OPEN AOS model?

Detailed explanations of outcomes to be measured across all three components are presented in Additional file 2.

### Component 1: Comparison of OPEN AOS model to standard care

#### Design

**Fig. 1:**
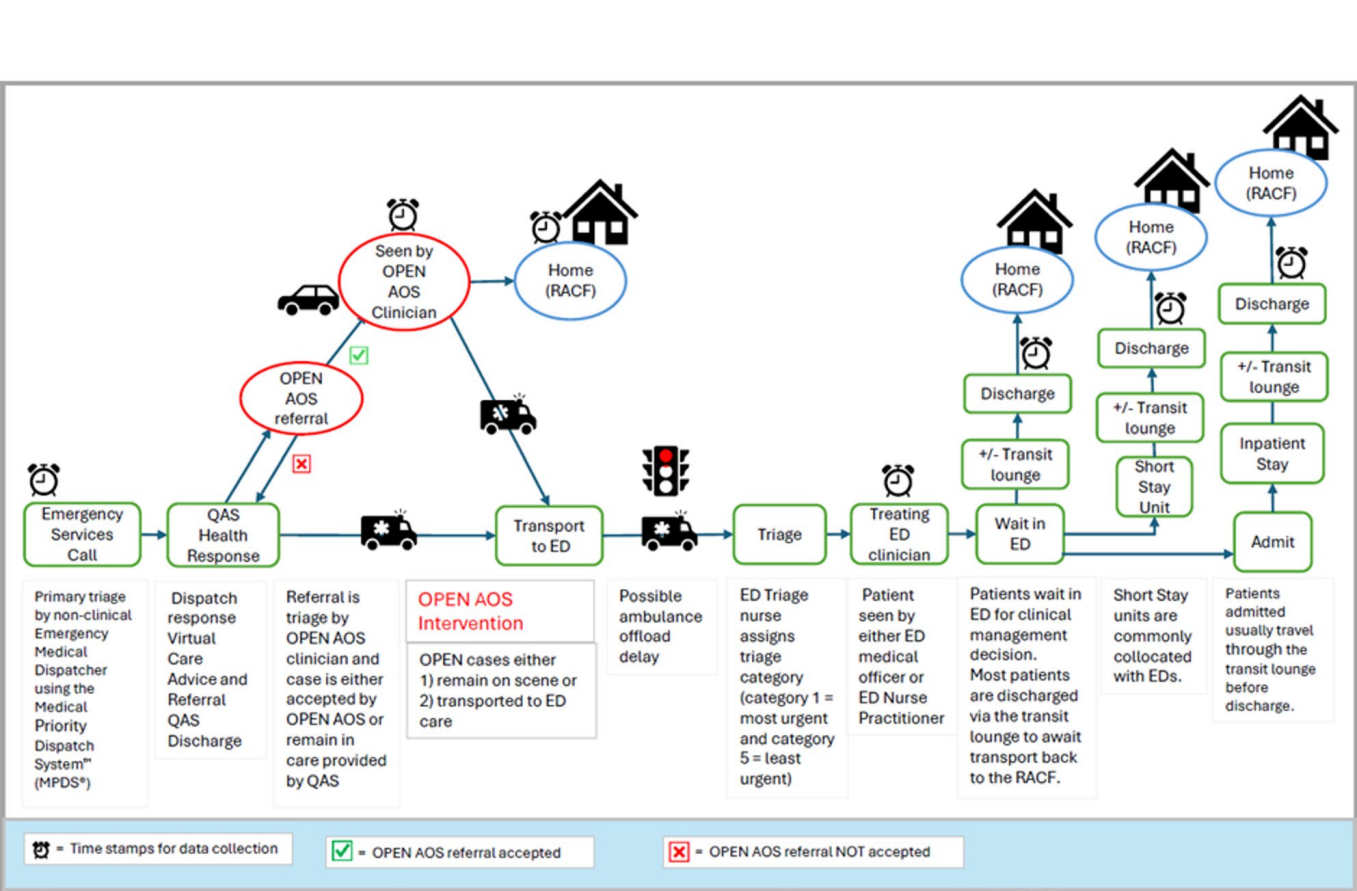
OPEN AOS model patient journey following referral via emergency services

Component 1 is a retrospective, quasi-experimental design. This design was considered to be the most appropriate given the availability of pre-existing data and feasibility [48, 49]. Individual and treatment characteristics and outcomes for patients treated by OPEN AOS (intervention group) or received standard care (control group) will be compared. Figure 1 illustrates the potential patient pathways from the RACF, depicting management either by OPEN AOS or via ambulance attendance with transport to the emergency department (ED).

### Samples

In the 12-month study period from 1 July 2022 to 30 June 2023, approximately 7600 ED presentations from RACFs arrived by ambulance to district’s ED sites. During that same period, 735 patients were treated by OPEN AOS during their operational hours (08:00–20:00) Monday to Sunday. Patients treated in the Intervention group will be matched with up to three randomly selected control group patients without replacement with matching on age (+/- 5 years), and exact matching on sex and diagnostic classification section (or chapter if diagnoses are uncommon) as per the International Classification of Diseases, 10th Revision Australian Modification (ICD-10-AM) using the ‘R’ package Matching: Multivariate and Propensity Score Matching with Balance Optimization [50]. Groups sizes of 351 for the intervention group and 1053 for the control group will achieve 90% power to reject the null hypothesis of no effect on the time to be seen by treating clinician when there is a Cohen’s d small effect size of 0.2 using a two-sided, two-sample test assuming equal variance and an alpha of 5% [51].

### Inclusion criteria

Intervention: Adults aged 65 years or older who are permanent residents of RACFs situated within the MNHHS district geographical catchment area, with acute care needs and who were referred by the ambulance service to OPEN AOS between 08:00 and 20:00, Monday to Sunday, from 1 July 2022 to 30 June 2023. Patients can receive treatment by OPEN AOS on more than one occasion within the study period.

Control: Adults aged 65 years or older who are permanent residents of RACFs situated within the MNHHS district geographical catchment area, who received standard ambulance attendance and transport to a MNHHS ED between 08:00 and 20:00, Monday to Sunday, from 1 July 2022 to 30 June 2023. Only the first presentation for each patient within the study period will be examined.

### Exclusion criteria

Patients were excluded if they (1) were assigned emergency status at primary (or secondary) triage (for any clinical reason); (2) did not reside in an RACF or resided in respite care; (3) were attended by ambulance and transported to ED care outside of OPEN AOS model operational hours (i.e. before 08:00 or after 20:00 Monday to Sunday); (4) were referred to OPEN AOS by any referrer other than ambulance services; or (5) received care outside the designated study period (1 July 2022 to 30 June 2023). (6) Patients included in the intervention group, i.e. those seen by OPEN AOS will be excluded from the control group.

### Data collection and linkage

Patients living in RACFs are identified upon arrival to ED within EDIS^®^ and recorded as arriving by ambulance from a nursing home. Data extraction will occur retrospectively from state-based and MNHHS databases. An anonymous unique identifier (linkage ID) will be assigned to all cases by the Queensland Health Statistical Service Branch (SSB), enabling linkage of data from all sources (Table 1). These data provided to the researchers will not contain any key patient identifying features such as Unit Record Number (URN), name, address or Date of Birth (DOB). However, data such as age, postcode, and health facility will be included in the collection.

**Table 1:** Data sources, date ranges and data descriptions for the OPEN AOS evaluation study

Data Source	Time Period	Data Description
RADAR Registry	1 Jul 2022 to 30 Jun 2023	OPEN AOS activity plus outcome data including: • outcomes of intervention • time stamps • clinician delegation Patient demographics for matching variables
Emergency Department Information System (EDIS^®^)	1 Jul 2022 to 28 Jul 2023	ED presentations of cohort members at one of the participating hospitals. • outcomes of intervention • time stamps Patient demographics and RACF address (presentation postcode and suburb) for matching variables
Metro North Health Funding and Data Insights	1 Jul 22 to 28 Jul 2023	Costs of initial ED presentations and subsequent re-presentations within 48 h and 28 days Patient unit record number for matching variables
Queensland Hospital Admitted Patient Data Collection	1 Jul 2020 to 30 Jun 2023	Patient co-morbidities identified during any hospital admission (using ICD-10-AM codes and ICD-10-AM code chapters), during the 24-month period preceding their presentations to the ED Patient demographics for matching variables
Death Registrations from the Registry of Births, Deaths and Marriages Queensland Cause of Death Unit Record File from the Australian Coordinating Registry	1 Jul 2022 to 02 Jul 2023	Death within 48 h of discharge from either emergency department presentation or OPEN AOS intervention Patient demographics for matching variables
RiskMan Queensland Health’s system for recording and managing clinical incidents, workplace events, consumer feedback, and risk	1 Jul 2022 to 02 Jul 2023	Patient Clinical Incident data within 48 h of discharge from the ED Patient demographics for matching variables
Queensland Ambulance Service Clinical & Dispatch Data (DARF and CAD)	1 Jul 2022 to 30 Jun 2023	Queensland Ambulance Service data including; • ambulance dispatch response variables • case cycle times (or time stamps) Patient demographics and clinical care variables for matching

*CAD* Computer Assisted Dispatch, *DARF* Digital Ambulance Report Form, *ED* Emergency Department, *ICD-10-AM* International Classification of Diseases, 10th Revision Australian Modification, *OPEN AOS* Older Persons Emergency Service Acute Outreach Service, *RADAR* Residential Aged Care District Assessment and Referral

### Data analysis

For our analysis, the comparison of interest is between the intervention and control groups. Re-presentations by patients in either group are not considered as independent events and will be excluded from analyses, however the number of re-presentations during the study period will be noted for the OPEN AOS group. Categorical data will be presented as frequency and percentages, while continuous data will be reported as mean with standard deviation or median with interquartile ranges, depending on the distribution of the data. Categorical data will be analysed using the Pearson Chi-squared test or Fisher’s exact test, and continuous variables using the Student t-test or Mann-Whitney U test. Time to treating emergency clinician and time to discharge from the episode of care will be examined using a generalised linear model or parametric regression model if events are censored e.g., death of a patient. ED re-presentation and mortality will be examined using logistic regression if there are sufficient events. All regression models will be multivariable with fixed effects for triage score, weekend presentation, season, and ambulance referral time. The inclusion of Charlson Comorbidity Index [52] in regression analyses will depend on the extent of missing data. Component 1 will be reported in accordance with the Enhancing the QUAlity and Transparency Of health Research (EQUATOR) guidelines for reporting non-randomised studies [53].

### Component 2: health economic evaluation

Component 2 will use a decision analytic model to estimate the cost-efficiency of the OPEN AOS model intervention with standard care, taking into consideration the subsequent probability of presentation and re-presentation to the ED. The perspective for the analysis will be from the public hospital and health system.

This economic evaluation will be undertaken using retrospective data collected concurrently with Component 1, drawing on the same sample, time periods, and a sub-set of the routinely collected de-identified and linked data. The primary outcome will be the incremental expected cost per patient treated via OPEN AOS compared to standard care. Component 2 will follow the Consolidated Health Economic Evaluation Reporting Standards 2022 [54].Total annual cost of the OPEN AOS and additional ED equivalent capacity created will be based on forecast annual utilisation of OPEN AOS derived from historic utilisation estimates. An estimate of the net monetary benefit of the OPEN AOS model will be derived based on the annual utilisation of the OPEN AOS model multiplied by the incremental expected cost (saving) per person seen by the OPEN AOS model.

### Data analysis

Costs include additional resources to deliver the new model of care (staffing and non-staffing costs) and downstream costs, such as ED presentation and ambulance transport re-deployment. ED costings will be based on an official average ED cost for the state of Queensland [55]. QAS costs will be based on average cost per ambulance incident for MNHHS over the study period [56]. Costs for the standard care will be based on the cost of QAS and ED presentation, considering subsequent ED re-presentation as provided from Component 1. As in component 1, all patient level data will be retrospectively sourced from existing routine data collection systems and linked by the state-based Statistical Services Branch. Uncertainty will be characterised using one way sensitivity analysis and probabilistic sensitivity analysis using Monte Carlo simulation [57, 58]. Furthermore, annualized cost, annual additional capacity created with respect to ED presentations avoided, and annual cost savings will be modelled for all 87 RACFs.

### Component 3: structure and process evaluation of OPEN AOS

#### Design

Component 3 uses a qualitative design and will involve conducting focus groups and semi-structured interviews to better explore and understand contextual factors influencing implementation, and potential scale and spread of the OPEN AOS model. Factors impacting implementation, scale and spread have been adapted from the PRISM framework and include elements such as the organisation and patients’ perspectives of the intervention; characteristics of the patients and the organisation; the external environment; and infrastructure to support implementation and sustainability [59].

### Participants

Four groups will be invited to participate in this qualitative component:

11. OPEN AOS staff members (*n* ≤ 20). Participants will be currently employed by MNHHS and have worked in the OPEN AOS model for at least 6 months.
12. Older adults living in RACF and/or their family members who have received care from the OPEN AOS team (*n* ≤ 20). Participants will have had personal experience or family member experience of their relative being cared for by the OPEN AOS team within the six weeks preceding the interview.
13. Staff from Queensland Ambulance Service, local Residential Aged Care District Assessment and Referral (RADAR) teams, RACFs and GPs who have referred patients to the OPEN AOS team (*n* ≤ 20).
14. Executive and management stakeholders (*n* ≤ 10). Senior decision-makers involved in policy, funding, collaboration or service design related to OPEN AOS and referrers to OPEN AOS.

Participants will be invited via the following methods:

- Emails will be sent to local RADAR, OPEN AOS staff and executive stakeholders via the OPEN AOS Nursing Director and/or Clinical Director. A research team member will attend RADAR and OPEN AOS staff meetings to advertise the study and invite participation.
- QAS will facilitate the invitation of ambulance staff including management and ambulance officers via their usual email channels. Interested staff members will be invited to contact the research team at which time they will be provided an information sheet and consent form.
- An email will be sent to the GP’s of RACF residents who received care from the OPEN AOS to advertise and invite them to participate in the study.
- RACF residents and/or their attending family members will be invited by the treating clinician at the conclusion of an OPEN AOS intervention, and asked if they would like to be contacted by the research team for more information about the study. Residents with cognitive impairment will be identified by research staff through review of clinical notes or on discussion with next of kin. The participant will have their Enduring Power of Attorney contacted and a dual consent process will occur to ensure understanding of the risks and benefits of participation.

All interested individuals will be provided with an information sheet and consent form. Invited individuals will be provided the opportunity to ask and have questions answered before written consent is obtained and the interview or focus group is conducted. Convenience sampling will be used to obtain information-rich cases from older adults who have experienced the OPEN AOS model. Some participants may be invited to take part in both a focus group and a semi-structured interview. This dual participation is intended to enhance data richness by capturing both collective perspectives and individual experiences. Participation in both activities is voluntary, and specific participants will be informed that they may choose to participate in one or both. We will predominantly conduct focus groups for participants in similar roles or within teams, and semi-structured interviews for patients and families to ensure privacy and comfort. Semi-structured interviews will also be used for GPs and executives for easier access and scheduling (up to 70 participants in total). A detailed description of the planned qualitative data collection methods is provided in Additional file 3.

### Data collection

Semi-structured interviews will be scheduled according to participant preference, with options for in-person or online participation. Similarly, focus groups will be arranged at times and in formats that maximise participation, offering both in-person and online attendance. All semi-structured interviews and focus groups will be audio-recorded. Participants will be invited to respond to semi-structured questions guided by an a priori system for categorising qualitative data proposed by Bogdan and Biklen [60]. These a priori categories will be adapted from the PRISM framework [44] and shaped by the contextual characteristics relevant to the evaluation of the OPEN AOS model. Registered nurses recruited specifically as research assistants and the lead investigator who are not OPEN AOS clinicians will conduct the semi-structured interviews and focus groups to minimise bias. An iterative approach will be adopted, with earlier interviews shaping edits to the interview guide for later interviews. If participant distress is noted, the interviewer will ask if they wish to cease the interview or focus group and will provide a pamphlet on appropriate services to contact for assistance.

Recorded interviews and focus groups will be transcribed and anonymised prior to analysis. To achieve data saturation, data collection will continue until no new themes relevant to the study emerge from successive interviews. This will be achieved through ongoing data analyses [61]. Based on prior literature [62], we anticipate saturation may occur within approximately 12 interviews for a relatively homogeneous participant group; however, final sample size will be guided by the principle of saturation rather than a predetermined number.

### Data analysis

The qualitative data will be analysed using thematic analysis, following the six-step approach described by Braun and Clarke [63] and informed by a priori categorisation to guide the coding process whilst remaining open to inductive codes. This will ensure that interview and focus group transcripts are analysed in alignment to the research question based on PRISM domains [44]. Transcripts of interviews and focus groups will be read and re-read with initial tags assigned to sections of text to familiarise the researchers with the data. Using a priori labels provides a separate accounting scheme for the deductive analysis of qualitative data by limiting analysis to focus on contextual characteristics experienced by participants relevant to the OPEN AOS model. The data collected from the four groups of interviewees will be analysed separately to compare and contrast findings [60]. NVivo will be used to manage and organise the transcribed interview data and coding system, supporting systematic coding, retrieval and comparison. Component 3 will follow the Consolidated criteria for reporting qualitative research (COREQ) reporting guideline for qualitative research [64].

### Rigour

To ensure the trustworthiness and credibility of findings in Component 3, several strategies aligned with Lincoln and Guba’s (1985) framework for qualitative rigour will be implemented [62]. First, the standardisation of coding through use of a priori categories will provide a consistent framework for analysing data. Additionally, three research team members will code the data independently and discuss preliminary findings with the research team, debating any differences and agreeing by consensus. This approach enhances the reliability and dependability of the analysis. To minimise bias, the interviewers will use a reflexive approach, adhere to a structured interview guide, and avoid influencing participant responses. To ensure transferability we will include sufficient descriptive data such as verbatim quotes, to offer rich contextual insights, allowing readers to assess the applicability of findings to other contexts. Finally, triangulation of data by incorporating multiple perspectives from the OPEN AOS team, referrers and patients and/or their carers will strengthen credibility and minimize potential bias.

## Discussion

The increase in patient volumes and more diverse emergency care needs of communities have seen a need to shift from traditional ED designs to designs better able to address safety, quality, and patient experience expectations [65]. Further shifts have occurred and a recent (2024) systematic review on strategies for improving ED-related outcomes of older adults has concluded that efforts to improve older patients’ needs should focus on interventions initiated outside the ED [66]. Novel models of care have and continue to emerge that utilise alternative care pathways to reduce unnecessary ambulance transport to hospital, ED investigations and interventions. The OPEN AOS model represents an innovative ED substitution approach, whereby person-centred emergency care is delivered to older adults living in RACFs.

### Limitations

This study is subject to several methodological limitations. First, the findings should be interpreted in the context of the limitations inherent to non-randomised comparisons. Without random allocation, there is an increased risk of selection bias, baseline differences, and unmeasured confounders that may influence observed outcomes. These factors limit the ability to draw causal inferences and should be considered when evaluating the study’s conclusions. We envisage that findings from this study will inform a future randomised controlled trial. Second, the recruitment strategy may have introduced recruitment bias. The approaches adopted may favour individuals who were more engaged with organisational communication channels, more available, or more positively oriented toward the service, potentially underrepresenting those with differing experiences or lower levels of engagement.

### Future implications

This study will provide ED clinicians and policy makers with evidence on the impact, translatability and cost efficiency of the OPEN AOS model within one hospital and health service in South-East Queensland, Australia. Findings will inform local model refinements and guide future resource allocation decisions. Importantly, this evidence will add to the body of knowledge regarding emergency care for older vulnerable adults residing in RACFs. This study will provide valuable information regarding this emergency outreach model’s capacity to deliver timely care and reduce unnecessary transport of older adults to ED. Furthermore, the identification of barriers and enablers to implement and scale effective emergency substitution models can guide future endeavours to strengthen emergency care for older adults.

Findings from this study have the potential to inform national and international frameworks aimed at improving access to and quality of emergency care for older adults. These insights may guide adaptation of similar models in other jurisdictions and support the development of patient-centred approaches to emergency care for vulnerable older populations. As the global community adapts to an aging population, continued efforts to optimise care for older adults in their place of residence through the use of alternative care options (such as OPEN AOS) are required.

## Supplementary Information

Supplementary Material 1.

Supplementary Material 2.

Supplementary Material 3.

## Data Availability

No datasets were generated or analysed during the current study.

## Abbreviations

COREQ: Consolidated criteria for reporting qualitative research
DOB: Date of birth
ED: Emergency departments
EDIS: Emergency department information system
EQUATOR: Enhancing the quality and transparency of health research
FTE: Full-time equivalent
GP: General practitioner
HBCIS: Hospital based corporate information system
HREC: Human research ethics committee
ICD-10AM: International classification of diseases, 10th revision Australian modification
MNHHS: Metro north hospital and health service
NHMRC: National health and medical research council
NP: Nurse practitioner
OPEN AOS: Older persons emergency network acute outreach service
PRISM: Practical implementation sustainability model
QAS: Queensland ambulance service
QHAPDC: Queensland hospital admitted patient data collection
RACF: Residential aged care facility
RADAR: Residential aged care assessment and referral
REAIM: Reach, effectiveness, adoption, implementation and maintenance
SMO: Senior medical officer
SSB: Statistical services branch
URN: Unit record number

## Footnotes

1 NPs are advanced practice registered nurses with expert knowledge, complex decision-making skills and clinical competencies who have completed an approved master’s degree education program [40, 41].

2 NPCs practice under supervision of an experienced NP or SMO and is an experienced RN who is completing required approved training and education to become a NP [40].

3 RNs have completed a program of study which meets the RN standards of practice and whose scope of practice includes critical analysis in the planning, managing and evaluation of health care [40].

4 A SMO is registered with the Medial Board of Australia, has extensive post registration clinical practice experience and is able to manage complex cases independently whilst maintaining knowledge and skills through ongoing education and training. SMOs must be credentialed and have a defined scope of clinical practice, ensuring they deliver safe, high-quality care [42, 43].

5 Senior pharmacists provide and are individually accountable for the delivery of high-level clinical pharmacy services in accordance with prescribed professional and ethical standards with minimal direct supervision [67].

6 Diagnostic classification as per the International Classification of Diseases, 10th Revision Australian Modification (ICD-10-AM).

7 Clinical Incidents include events or circumstances that resulted, or could have resulted, in unintended and/or unnecessary harm to a patient/consumer; and/or a complaint, loss or damage.
