# Supplementary material for: A pragmatic implementation and outcomes evaluation of the older persons emergency network acute outreach service (OPEN AOS) model utilising the integrated PRISM and RE-AIM framework: the OPEN AOS study protocol
Source: BMC Geriatr. 2026 Jan 13;26:344. doi: 10.1186/s12877-025-06917-2 (PMC12990526; doi:10.1186/s12877-025-06917-2)
Supplement: Supplementary file 1 — Supplementary Material 1. [file 12877_2025_6917_MOESM1_ESM.pdf]

Supplementary File 2: Table S2. Clinical and administrative responsibilities of OPEN AOS team members

Role	Clinical Responsibilities	Administrative Responsibilities
OPEN AOS Nurse Practitioner (NP) and Nurse Practitioner Candidate* (NPC)	Provide ED substitutive care via outreach, telehealth or phone; liaise with SMO; demonstrate autonomy and accountability; provide specialist nursing interventions (e.g., prescribing, ordering pathology); advanced clinical support and leadership.	Maintain effective communication; provide clinical leadership and mentorship; evaluate service outcomes; participate in data collection and reporting.
OPEN AOS Registered Nurse (RN)	Collaborate with all staff; demonstrate clinical skills relevant to acute care outreach; pre/post RACF visit communication; ensure accurate data input.	Commitment to continuous quality improvement; portfolio involvement; participate in data collection and reporting.
OPEN AOS Pharmacist	Deliver specialist clinical pharmacy services; conduct comprehensive medication reviews; consult on medication management and storage.	Provide professional leadership in pharmacy practices; lead implementation of pharmacy-related initiatives; provide education to RACF staff and GPs; support RADAR clinicians.
OPEN AOS Senior Medical Officer (SMO)	Provide clinical leadership, supervision, and governance; telephone support for GPs and QAS; documentation and communication of planned care; consult on delegation activities.	Implement model of care; promote ED substitution; lead change management strategies; facilitate interdisciplinary discussions; collaborate on research relationships.

Footnotes: ED = Emergency Department; RACF = Residential Aged Care Facility; GP = General Practitioner; QAS = Queensland Ambulance Service; RADAR = Residential Aged Care District Assessment and Referral.

*Nurse Practitioner Candidates can perform all NP responsibilities but may require supervision by NP or SMO depending on their level of experience.
