# Supplementary material for: A pragmatic implementation and outcomes evaluation of the older persons emergency network acute outreach service (OPEN AOS) model utilising the integrated PRISM and RE-AIM framework: the OPEN AOS study protocol
Source: BMC Geriatr. 2026 Jan 13;26:344. doi: 10.1186/s12877-025-06917-2 (PMC12990526; doi:10.1186/s12877-025-06917-2)
Supplement: Supplementary file 2 — Supplementary Material 2. [file 12877_2025_6917_MOESM2_ESM.pdf]

Additional file 2: Table S2. Research questions and outcomes

**Component 1 Research Question:** To what extent (if any) do patients characteristics (such as age, sex, and diagnostic classification) and outcomes differ between OPEN AOS care and standard care (i.e. ambulance attendance with transport to ED care)?
**Outcomes:**<br>a. **Primary Outcome:**<br>Time to be seen by treating clinician, defined as the amount of time in minutes from the initial contact with QAS to the time seen by either:<br>I. the OPEN AOS clinician (intervention group); or<br>II. the ED medical officer (control group).<br><br>b. **Secondary Outcomes:**<br>I. Length of care episode, defined as the amount of time in hours from initial contact with QAS to discharge of care from either:<br>i. OPEN AOS (on scene); or<br>ii. ED (if transported to ED including time spent in a short stay unit and subsequently discharged); or<br>iii. Hospital (if admitted and subsequently discharged); or<br>iv. Death.<br>II. Ambulance transport to ED care: defined as the proportion of OPEN AOS patients requiring transport to ED for definitive care.<br>III. ED representation for any cause within 48 hours and 28 days of discharge from either an ED presentation or an OPEN AOS intervention.<br>IV. Death within 48 hours of discharge from either an ED presentation or an OPEN AOS intervention.<br>V. Clinical incident[7] data within 48 hours of discharge from either an ED presentation or an OPEN AOS intervention.
**Component 2 Research Question:** What contextual factors impacted implementation and future scale and spread of the OPEN AOS model?
Specifically:<br>a. What factors pertaining to the OPEN AOS model affected day-to-day operation of ambulance service, RACF, GP, local RADAR service and how?<br>b. What organisational characteristics are required to deliver the OPEN AOS model?<br>c. What are the experiences of ambulance officers, RACF staff, GPs and OPEN AOS and RADAR clinicians regarding OPEN AOS model processes?<br>d. What are patients' and carers' experiences with the OPEN AOS model?

**Component 3 Research Question:** Compared to standard care, what is the incremental cost (saving) per person of the OPEN AOS model?
Specifically:<br>a. What is the expected cost per presentation for the OPEN AOS model?<br>b. What is the frequency of an OPEN AOS presentation requiring an ED presentation?<br>c. What is the expected cost offset associated with avoiding an ED presentation?<br>d. What is the annual cost associated with OPEN AOS; additional ED equivalent capacity created and net monetary benefit of the OPEN AOS model?

[6] Diagnostic classification as per the International Classification of Diseases, 10th Revision Australian Modification (ICD-10-AM)

[7] Clinical Incidents include events or circumstances that resulted, or could have resulted, in unintended and/or unnecessary harm to a patient/consumer; and/or a complaint, loss or damage.
