# Supplementary material for: A pragmatic implementation and outcomes evaluation of the older persons emergency network acute outreach service (OPEN AOS) model utilising the integrated PRISM and RE-AIM framework: the OPEN AOS study protocol
Source: BMC Geriatr. 2026 Jan 13;26:344. doi: 10.1186/s12877-025-06917-2 (PMC12990526; doi:10.1186/s12877-025-06917-2)
Supplement: Supplementary file 3 — Supplementary Material 3. [file 12877_2025_6917_MOESM3_ESM.pdf]

Supplementary file 3: Table S3. Overview of Qualitative Data Collection Methods

Interviewee Group	Data Collection Method	Interviewer	Rationale for Choice of Interviewer
Patients and/or carers	Semi-structured interviews	Coordinating Researcher or Research Assistant	Individual interviews ensure privacy and comfort when discussing personal care experiences.
RACF staff	Semi-structured interviews and focus groups	Coordinating Researcher or Research Assistant	Group discussions may facilitate shared reflection on implementation experiences within facility settings.
General practitioners	Semi-structured interviews	Coordinating Researcher or Research Assistant	Individual interviews are preferred due to scheduling challenges and the independent nature of GP practice.
RADAR representatives	Semi-structured interviews and focus groups	Research Assistant	The Research Assistant has no prior relationship with the RADAR team, reducing potential for social desirability bias.
QAS clinicians	Semi-structured interviews and focus groups	Coordinating Researcher or Research Assistant	Group format may promote discussion of shared operational experiences; interviewer assigned based on availability.
OPEN clinicians	Semi-structured interviews and focus groups	Research Assistant	The Research Assistant does not have an existing working relationship with participants, supporting perceived neutrality and reducing potential bias.

Executive/Management representatives (RACF, QAS, OPEN)	Semi-structured interviews	Coordinating Researcher	The Coordinating Researcher's understanding of system-level processes enables more informed and probing exploration of organisational and strategic factors.

Footnotes: RACF = Residential Aged Care Facility; QAS = Queensland Ambulance Service; OPEN = Older Persons Emergency Network.
